# Functional connectivity between the nucleus accumbens and amygdala underlies avoidance learning during adolescence: Implications for developmental psychopathology

**DOI:** 10.1017/S095457942400141X

**Published:** 2024-09-26

**Authors:** Benjamin M. Rosenberg, João F. Guassi Moreira, Adriana S. Méndez Leal, Natalie M. Saragosa-Harris, Elizabeth Gaines, Wesley J. Meredith, Yael Waizman, Emilia Ninova, Jennifer A. Silvers

**Affiliations:** 1 Department of Psychology, University of California, Los Angeles (UCLA), Los Angeles, CA, USA; 2 Department of Psychology, University of Southern California, Los Angeles, CA, USA; 3 College of Social Work, Florida State University, Tallahassee, FL, USA

**Keywords:** Adolescence, anxiety, depression, functional connectivity, threat learning

## Abstract

**Background:**

Reward and threat processes work together to support adaptive learning during development. Adolescence is associated with increasing approach behavior (e.g., novelty-seeking, risk-taking) but often also coincides with emerging internalizing symptoms, which are characterized by heightened avoidance behavior. Peaking engagement of the nucleus accumbens (NAcc) during adolescence, often studied in reward paradigms, may also relate to threat mechanisms of adolescent psychopathology.

**Methods:**

47 typically developing adolescents (9.9–22.9 years) completed an aversive learning task during functional magnetic resonance imaging, wherein visual cues were paired with an aversive sound or no sound. Task blocks involved an escapable aversively reinforced stimulus (CS+_r_), the same stimulus without reinforcement (CS+_nr_), or a stimulus that was never reinforced (CS−). Parent-reported internalizing symptoms were measured using Revised Child Anxiety and Depression Scales.

**Results:**

Functional connectivity between the NAcc and amygdala differentiated the stimuli, such that connectivity increased for the CS+_r_ (*p* = .023) but not for the CS+_nr_ and CS−. Adolescents with greater internalizing symptoms demonstrated greater positive functional connectivity for the CS− (*p* = .041).

**Conclusions:**

Adolescents show heightened NAcc-amygdala functional connectivity during escape from threat. Higher anxiety and depression symptoms are associated with elevated NAcc-amygdala connectivity during safety, which may reflect poor safety versus threat discrimination.

## Introduction

Adolescence is characterized by increasing autonomy and pursuit of novel experiences, corresponding with heightened behavioral and cognitive flexibility (Crone & Dahl, 2012). During adolescence, changing environmental demands coincide with neurodevelopmental shifts that motivate exploration of one’s environment, while still protecting survival. Prevailing theories in developmental affective neuroscience and developmental psychopathology point to changes in both reward and threat circuitry during adolescence as being, respectively, important for approach and avoidance motivations, and more generally for healthy development and well-being (Baker & Galván, 2020; Gee et al., 2018; Silk et al., 2012). However, relatively little research has looked at how these systems may work together to support adaptive associative and instrumental learning processes. The present study aims to (1) identify interactions between canonical threat and reward neurocircuitry during aversive learning and (2) explore potential links to emerging symptoms of anxiety and depression during adolescence.

### Threat and safety learning

Threat and safety learning are considered central to the etiology and treatment of anxiety disorders, which are known to emerge or worsen during adolescence (Baker & Galván, 2020; Shechner et al., 2014). Relatively young children can learn cues (i.e., conditional stimuli; CSs) that predict threat or safety and provide a foundation for emerging threat appraisals (Britton et al., 2011). However, growing evidence suggests that the ability to discriminate between dangerous and safe CSs emerges continues to improve across childhood (Grasser & Jovanovic, 2021; Jovanovic et al., 2014), throughout adolescence (Reinhard et al., 2022), and into adulthood (Lau et al., 2011). Furthermore, preliminary evidence suggests that adolescents tend to show less differentiation of threatening and safe stimuli than adults, corresponding with heightened avoidance of safe stimuli and overgeneralization of fear to perceptually similar stimuli (Klein et al., 2021). There is mixed evidence regarding age-related differences in fear extinction (potentially attributable to methodological differences, see Stenson et al., 2023), with some studies suggesting that adolescents compared with adults tend to exhibit attenuated fear extinction (Haddad et al., 2011; Linton & Levita, 2021; Pattwell et al., 2012; for a review, see Baker et al., 2014), and others suggesting no difference between adolescents and adults in fear extinction (Abend et al., 2020; Waters et al., 2017) or fear reinstatement (Den et al., 2015; Waters et al., 2017). Collectively, these results suggest that the ability to discriminate threat versus safety undergoes continued refinement throughout the adolescent period.

Neuroimaging studies of threat learning in adults have highlighted roles for the amygdala (though amygdala findings are more mixed, see Fullana et al., 2016), dorsal anterior cingulate cortex (dACC), anterior insular cortex, and ventromedial prefrontal cortex (vmPFC) during fear acquisition (Battaglia et al., 2020; Etkin & Wager, 2007; Fullana et al., 2016; Greco & Liberzon, 2016; Milad et al., 2014; Sehlmeyer et al., 2009), as well as the amygdala, hippocampus, and vmPFC in fear extinction (Greco & Liberzon, 2016; Milad et al., 2014). Relatively few neuroimaging studies of Pavlovian fear processes have been published in adolescents (Treanor et al., 2021). However, it has been suggested that differences in fear learning observed in adolescents versus adults may be attributable to adolescents possessing relatively mature threat neurocircuitry (emphasizing the amygdala) in tandem with less mature prefrontal cortex (Grasser & Jovanovic, 2021; Morriss et al., 2019). For example, adolescents compared with adults tend to show less activation of the vmPFC and dlPFC during fear extinction recall (Ganella et al., 2018).

### Adolescent exploration, reward pursuit, and risk-taking

Exploration and reward pursuit are considered hallmarks of adolescence that have the potential to promote both adaptive and maladaptive behaviors, depending on context (Ciranka & van den Bos, 2021; Duell & Steinberg, 2019; Romer et al., 2017). Reward-related activation of the nucleus accumbens (NAcc), a region of the ventral striatum that is responsible for dopaminergic signaling across a variety of tasks and motivated behaviors (Floresco, 2015; Nicola, 2007), has been shown to peak during adolescence (Braams et al., 2015; Cohen et al., 2010; Schreuders et al., 2018; Silverman et al., 2015; Urošević et al., 2012). A wealth of prior studies has focused on how increasing striatal engagement coincides with increasing reward pursuit and sensitivity, thereby steering adolescents toward greater engagement in specific exploratory behaviors that promote new learning, including risk-taking (Barkley-Levenson & Galván, 2014; Depasque & Galván, 2017; Galván et al., 2006; Galván, 2010; Somerville et al., 2010; Van Duijvenvoorde et al., 2016; Walker et al., 2017). Reinforcement learning rates are higher among adolescents than adults (Topel et al., 2023) and increase throughout adolescence (Master et al., 2020), and this is particularly evident in paradigms involving rewards (Palminteri et al., 2016) or positive feedback (Davidow et al., 2016). Collectively, these studies highlight adolescence as a period characterized by elevated recruitment of reward neurocircuitry and corresponding elevations in reward sensitivity, thereby establishing a basis for learning and a propensity for risk-taking.

### Threat and reward: an integrated perspective

While in everyday life reward and threat neurocircuitry ostensibly work closely together to support adaptive learning and decision making (e.g., to support approach and avoidance decisions, see Peris & Galván, 2021), they have been largely studied separately in adolescent samples (although some studies have evaluated threat and reward circuitry in relation to cognitive control of emotions, e.g., Heller et al., 2016). For example, the adolescent risk-taking literature has largely emphasized development of reward-related processes while ignoring threat processes (Baker & Galván, 2020). That said, some models of adolescent motivated behavior have integrated the development of threat neurocircuitry into conceptualizations of adolescent impulsivity and risk-taking behavior, noting a tendency for peaking NAcc recruitment to “tip” adolescent behaviors away from amygdala-mediated avoidance behaviors and toward risks (Ernst et al., 2006; Ernst, 2014; Richards et al., 2012).

Relatedly, it has been theorized that peaking striatal expression in adolescence may have implications for adolescent threat processing (Lago et al., 2017). Indeed, there is striking evidence that striatal neurocircuitry may be crucial for supporting avoidance of or escape from threats. For example, striatal dopamine signaling, a hallmark of reward prediction error (Schultz, 2013), has been tied to the processing of aversive or threatening stimuli (Brooks & Berns, 2013; França & Pompeia, 2023; McCutcheon et al., 2012; Verharen et al., 2020). More specifically, the NAcc is thought to coordinate with threat neurocircuitry (particularly the amygdala, hippocampus, and medial prefrontal cortex) to support fearrelated avoidance behaviors in both rodents and humans (for a review, see Wong et al., 2022). Similar neurocircuitry (e.g., amygdala, dACC, insula, NAcc, and vmPFC) is involved in studies of threat responding and defensive behaviors like freezing or escape (Mobbs et al., 2007, 2009, 2020). In humans, the NAcc has been shown to signal imminence of both rewards and threats (Murty et al., 2023). It has likewise been theorized that the vmPFC coordinates with the ventral striatum (including the NAcc) to assess the safety or threat of a given situation, and to make subsequent decisions about how to respond (Tashjian et al., 2021). In sum, there are compelling theoretical and empirical reasons to believe that striatal reward circuitry contributes to adaptive learning and responding to threats – for example, by coordinating escape or avoidance behavior, at least in adults. However, it is relatively unknown how these brain-behavior links change during adolescent development (França & Pompeia, 2023).

Other theoretical work suggests that positively valenced systems are inextricably linked to threat processing – for example, the removal of threat can be an inherently rewarding experience (for a review, see Rosenberg, Barnes-Horowitz, et al., 2024). More specifically, striatal dopamine signals the unexpected omission of aversive outcomes during Pavlovian fear extinction (Kalisch et al., 2019; Gentry et al., 2019; Salinas-Hernández & Duvarci, 2021) corresponding with subjective experiences of relief (Willems et al., 2023), a positive emotion that is experienced as similarly rewarding compared with monetary gains (Leng et al., 2023) and is moderated by reward sensitivity (Leng et al., 2022, 2024). The positively valenced experience of relief is thought to (1) reinforce escape from the predicted dangerous outcomes (unconditioned stimulus; US) or instrumental avoidance of the CS (Carver, 2009; Deutsch et al., 2015), and (2) coincide with prediction error learning when an anticipated fearful outcome surprisingly does not occur (i.e., during Pavlovian fear extinction) (Vervliet et al., 2017). Positive experiences of threat omission may further relate to other positive emotions, such as “thrill,” that may be particularly desirable among adolescents and may relate to peaking NAcc expression during this period (Spielberg et al., 2014). Additional research is needed to understand how reward and threat processes support one another, especially in adolescence.

### Adolescence and the emergence of psychopathology

Adolescence coincides with peak incidence rates across a range of internalizing psychopathology, including anxiety and depression (Kessler et al., 2007, 2012; Merikangas et al., 2010; Paus et al., 2008; Powers & Casey, 2015; Rapee et al., 2019). Aversive learning paradigms (e.g., Pavlovian fear acquisition) may offer a window into this phenomenon, as poor discrimination of threat and safety cues during development can lead to overuse of behavioral strategies that maintain or increase fear, such as avoidance (Waters & Craske, 2016). Likewise, individual differences in positive emotions are thought to moderate aversive learning during adolescence, suggesting that poor discrimination of threat and safety cues may also relate to emerging depression symptoms (e.g., anhedonia) (Rosenberg, Barnes-Horowitz, et al., 2024; Rosenberg, Young, et al., 2024).

Variations in the development of both threat and reward neurocircuitry may therefore be central to the onset of adolescent psychopathology (Baker & Galván, 2020; Xie et al., 2021; Zimmermann et al., 2019). For example, during late adolescence, heightened responsivity of threat neurocircuitry (particularly the vmPFC) during Pavlovian fear conditioning is associated with increases in anxiety-specific symptoms from late adolescence into young adulthood (Peng et al., 2023). Likewise, aberrations in reward sensitivity and associated neurocircuitry (e.g., low striatal recruitment, less striatal coordination with limbic regions) are associated with elevated risk avoidance behaviors among anxious adolescents (Baker et al., 2024; Galván & Peris, 2014; Peris & Galván, 2021). Considering potential interactions between these systems, individual differences in anhedonia symptoms (i.e., low reward sensitivity or motivation) are associated with (1) atypical patterns of brain activity during Pavlovian fear extinction (Young et al., 2021; Rosenberg et al., 2023) as well as (2) less acquisition of Pavlovian fear leading to greater generalization of fear to safe stimuli that persists into young adulthood (Rosenberg, Young, et al., 2024).

### The present study

The present study examined interactions between threat and reward neurocircuitry among typically developing adolescents during an aversive learning task involving three types of CS: a CS reinforced with an aversive sound (CS+_r_), an identical CS presented without reinforcement (CS+_nr_), or a different CS that was never reinforced (CS−). During CS+_r_ trials, participants were able to terminate the aversive sound (i.e., escape) by quickly pressing a button. We focused our analyses on the NAcc as well as regions that have been implicated in studies of threat learning (amygdala, vmPFC, hippocampus, insular cortex, dACC). Considering theories of how threat and reward neurocircuitry interact during the instrumental removal of threat (e.g., escape, avoidance) or unexpected omission of threat (e.g., fear extinction, threat prediction error) (Deutsch et al., 2015; Vervliet et al., 2017), and considering existing literature on the coordination between the NAcc and threat-related neurocircuitry among rodents or adult humans (e.g., Wong et al., 2022), we hypothesized that functional connectivity between the NAcc and threat neurocircuitry would be greater for stimuli associated with threat termination/omission (CS+_r_, CS+_nr_), and potentially greatest for the CS+_r_, compared with the stimulus involving no threat (CS−). Given that participants had not seen the stimuli prior to this task, and given the relatively short duration of task blocks, we hypothesized that differences among the blocks would emerge and be most notable during the final task blocks. Given prior research suggesting that anxiety and depression are associated with aberrances in safety processing and related neurocircuitry (Grasser & Jovanovic, 2021; Odriozola & Gee, 2021; Pittig et al., 2018), we additionally explored whether symptoms of anxiety and depression were associated with functional connectivity between the NAcc and threat neurocircuitry during the task.

## Materials and methods

### Participants

Participants were 47 typically developing individuals ages 9.9–22.9 years (24 female, mean age = 15.23 years, SD = 3.75 years). The sample included nine individuals ages 9.9–11.9 years old, 26 individuals ages 11.9–17.9 years old, and 12 individuals older than 18 years old. Demographics are summarized in Table 1. These participants were part of a broader longitudinal study investigating the impact of early life experiences on the neural bases of socioemotional development. Participants included in the current set of analyses were those who provided usable data from an fMRI scanning session and did not have a history of early social deprivation. All research was completed at the University of California, Los Angeles (UCLA) and was approved by the UCLA Institutional Review Board. All minor participants provided informed assent, and their parents provided informed consent, and all 18+ participants provided consent.

Two subjects were excluded for excess head motion (as described below, this was established as participants with > 20% of volumes having average framewise displacement exceeding 0.9 mm or global BOLD signal changes above 5 standard deviations), yielding a final sample of *n* = 45 subjects. Two subjects had incomplete CS+nr data and were therefore excluded from omnibus tests (*n* = 43). These subjects were included in multilevel models (*n* = 45) with incomplete CS+_nr_ blocks included as missing data.

### Aversive-learning task

**Figure 1.**
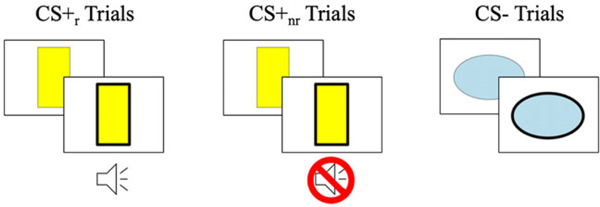
Visual representation of the aversive learning paradigm including three types of trial blocks: CS+_r_, CS+_nr_, and CS−. Participants completed eight total blocks (three CS+_r_, three CS+_nr_, two CS−).

Participants completed an aversive-learning task while inside of an MRI scanner. This task was identical to a version described elsewhere (see Silvers et al., 2016). Briefly, on each trial, participants viewed one of two shapes. Trials were organized into blocks in which a CS was reinforced with an aversive sound (CS+_r_), the same CS was not reinforced with an aversive sound (CS+_nr_), or a different stimulus was not reinforced with an aversive sound (CS−) (Figure 1). On each trial, the shape presented initially had a thin border that became thick after 1000ms. Participants were instructed to make a button response as soon as they saw the border of the shape thicken. During CS_r_ trials, at the same time that the border would begin to change, an aversive noise (US) started. Though participants were not told so, the button press terminated each trial and temporarily extinguished the US during CS_r_ blocks.

The US was a loud, metallic, high-frequency noise (Neumann et al., 2008) that was titrated for each participant before the task so that it was perceived as “annoying” but not painful (maximum volume, 92 dB). This calibration has been previously used in studies of aversive learning (e.g., Silvers et al., 2016). The US and CS+_r_ co-terminated when participants responded and another trial immediately began.

Participants completed eight 27 s task blocks (three CS+_r_ blocks, three CS+_nr_ blocks, and two CS − blocks) lasting 10–15 trials each (with exact length depending on RTs during a given block). Assignment of CS + and CS − to shape was counterbalanced across participants.

### Symptoms of anxiety and depression

Of the recruited sample, *n* = 36 parents of the included participants completed the Revised Child Anxiety and Depression Scales (RCADS-P; Chorpita et al., 2000). The RCADS-P is a 47-item questionnaire that probes anxiety symptomology (e.g., “All of a sudden my child will feel really scared for no reason at all”) and depression symptomatology (e.g., “Nothing is much fun for my child anymore”) with answer options ranging from 0 to 3 (0 = Never; 1 = Sometimes; 2 = Often; 3 = Always). The range of raw RCADS-P scores is 0–141, with higher scores suggesting greater levels of anxiety and depression. RCADS-P scores can be further separated into subscales including a 37-item anxiety subscale and 10-item major depression subscale. Total scores are converted to T-scores to enhance clinical utility and interpretability.

### fMRI data acquisition and analysis

#### Acquisition

Imaging data were acquired on a 3T Siemens Prisma scanner using a 32-channel head coil and a parallel image acquisition system (GRAPPA). A high-resolution T1-weighted, MPRAGE image was acquired for registration to functional runs (TR = 2400 ms, TE = 2.22 ms, flip angle = 8°, FOV = 256 mm^2^, 0.8 mm^3^ isotropic voxels, 208 slices). Functional images were acquired using a T2* EPI BOLD sequence. 33 axial slices were collected with a TR of 2000 ms and a 3 × 3 × 4 mm^3^ voxel resolution (TE = 30 ms, flip angle = 75°, FOV = 192 mm^2^). Participants completed the aversive-learning task by using a head-mounted mirror on the coil to view an LCD back projector screen.

#### Preprocessing

Before preprocessing, functional images were visually inspected for artifacts and biological abnormalities. No images contained obvious artifacts or biological abnormalities that warranted exclusion from further analysis. The scans for *n* = 2 subjects terminated prior to completion of the full task, yielding usable data for seven task blocks (i.e., incomplete third CS+_nr_ block). Incomplete blocks for these subjects were modeled as missing data during analyses (see below).

We implemented the default preprocessing pipeline for volume-based analyses in the CONN functional connectivity toolbox v22a (Whitfield-Gabrieli & Nieto-Castanon, 2012)^1^. Images were realigned using the default procedure in SPM12 (Andersson et al., 2001). We applied slice-timing correction for interleaved acquisition using the default procedure in SPM12 (Henson et al., 1999). Outlier volumes were identified and censored using the Artifact Detection Tools (ART) software in CONN applying the toolbox’s default settings (acquisitions with framewise displacement above 0.9 mm or global BOLD signal changes above 5 standard deviations). Data were then spatially normalized into the standard Montreal Neurological Institute (MNI) space (Friston et al., 1994), resliced to 2 mm × 2 mm × 2 mm voxels, and smoothed using a Gaussian kernel with a full-width at half-maximum (FWHM) of 6 mm.

#### First-level fMRI analyses

Six motion regressors (*x*, *y*, and *z* displacement; pitch, roll, and yaw rotation) and their first- and second-level derivatives were included as first-level covariates. Physiological noise was controlled with CompCor, an algorithm in which the timeseries of activation is extracted from subject-specific tissue masks (white matter, cerebrospinal fluid), and principal components analysis is applied to estimate physiological noise reflected in these timeseries, after which the resulting components are included as covariates in a denoising regression (for additional details on this approach, see Whitfield-Gabrieli & Nieto-Castanon, 2012). Finally, we applied a band-pass filter of 0.008–0.09 Hz to further remove high-frequency activity associated with physiological functioning and low-frequency activity associated with scanner drift.

#### Task performance

Task-based reaction times (RTs) were evaluated for each trial throughout the task. We calculated mean RTs for each participant during each task block. Mean RTs were winsorized for each task block, such that RTs below the 5^th^ percentile were replaced by RT values at the 5^th^ percentile, and RTs above the 95th percentile were replaced by RT values at the 95th percentile.

We conducted multilevel modeling in Stata 18.0 to test the main effect of Block (covarying for Age, Block, and Sex), the main effect of Stimulus Type (covarying for Age, Block, and Sex), and the interaction between Stimulus Type × Block in predicting RTs (covarying for Age and Sex). Models included random effects of the intercept and slope for each subject and fixed effects for Stimulus Type and Block as a within-subject factor. Stimulus Type was modeled as categorical variables in all analyses. We did not model Block as a continuous variable, as there were an unequal number of blocks for each stimulus type (i.e., such a model would estimate non-existent RT values for CS− Block 3). Therefore, Block was modeled as a categorical variable. Significance of the Stimulus Type × Block interaction was determined by comparing models with and without the interaction term included (using the *lrtest* function in Stata).

#### Functional connectivity

Using the Harvard-Oxford atlas, we extracted one ROI for bilateral NAcc as the seed region. We additionally extracted four ROIs for threat neurocircuitry (bilateral amygdala, bilateral hippocampus, bilateral insular cortex, and dACC). As the Harvard-Oxford atlas does not include a ROI for vmPFC, we used a 5 mm sphere centered on coordinates from a prior meta-analysis on Pavlovian fear learning (Fullana et al., 2016). These five ROIs were binarized and combined into a single ROI capturing the Threat Network. When significant results were identified, individual ROIs were analyzed separately in follow-up analyses (see below).

We conducted generalized psychophysiological interaction (gPPI) analyses (Friston et al., 1997) to examine NAcc connectivity with the Threat Network during the task. For each of the 8 task blocks, we computed beta weights (covarying for age, sex, and mean framewise displacement) for connectivity between the NAcc and each of the five target ROIs. We used a between-conditions omnibus contrast in CONN identifying any significant pairwise effects of task block to determine if connectivity between the NAcc and Threat Network was significantly associated with the task (*p*-unc < .05). Follow-up analyses evaluated the same contrast for each of the five ROIs within the Threat Network (*p*-unc < .05).

For significant target ROIs identified in the initial gPPI analysis, beta weights for each of the eight blocks were extracted from the CONN Toolbox and imported into Stata 18.0 for multilevel modeling of connectivity throughout the task. This analysis enabled a comparison across specific stimulus/block combinations. Using the same statistical procedure outlined above in *2.5.4 Task Performance*, we tested the interaction between Stimulus Type × Block in predicting connectivity betas (covarying for Age, Sex, and Mean Framewise Displacement).

#### Association with internalizing symptoms

For significant ROI results, follow-up analyses used multilevel modeling to evaluate the association between RCADS-P scores and gPPI beta weights for the stimuli across task blocks. Considering the reduced sample size of participants with RCADS-P data (*n* = 36), analyses focused principally on main effects of RCADS-P (covarying for Age, Stimulus Type, Block, and Sex) in predicting connectivity betas. We did not additionally analyze an Age × RCADS-P interaction due to power concerns and considering the exploratory nature of Age analyses (see below). Exploratory analyses also evaluated a RCADS-P × Stimulus Type interaction (covarying for Age, Block, and Sex). Similar analyses were also conducted to explore a main effect of RCADS-P or a RCADS-P × Stimulus Type interaction in predicting RTs during the task, as these behavioral results may provide added context for interpreting the neural data.

#### Exploratory analyses of age effects

As the ability to discriminate threat and safety tends to improve throughout adolescence, it is possible that functional connectivity between the NAcc and Threat Network becomes increasingly associated with threat discrimination as adolescents get older. Therefore, exploratory analyses tested the main effect of Age (covarying for Block, Stimulus Type, Sex, and Mean Framewise Displacement), Age × Stimulus Type interaction (covarying for Block, Sex, and Mean Framewise Displacement), and Age × Stimulus Type × Block interaction (covarying for Sex and Mean Framewise Displacement) in predicting connectivity betas for NAcc-ROI pairs significantly implicated in the main analyses. Similar analyses were also conducted to explore a main effect of Age (covarying for Stimulus Type, Block, and Sex), Age × Stimulus Type interaction (covarying for Block and Sex), and Age × Stimulus Type × Block interaction (covarying for Sex) in predicting RTs during the task, as these behavioral results may provide added context for interpreting the neural data.

#### Supplementary analyses

Exploratory analyses applied a seed-to-voxel approach to evaluate NAcc connectivity across the whole brain during the different task blocks (CS+_r_ > CS−, CS+_nr_ > CS−). These analyses are reported in the supplement. Furthermore, as the task is not optimized for a comparison of between-subjects differences in activation, we did not have a priori hypotheses about activation in this study. Univariate activation analyses are included in the supplement for ROIs implicated in significant functional connectivity results.

## Results

The aversive learning task involved blocks of CS+_r_, CS+_nr_, and CS− trials. We hypothesized that functional connectivity between the NAcc and threat neurocircuitry would be greatest for the CS+_r_ and CS+_nr_ compared with the CS− by the end of the task. For significant target ROIs, we additionally investigated associations between task functional connectivity and symptoms of anxiety and depression. Finally, we explored age effects to determine if significant target NAcc-ROI connectivity is increasingly associated with threat discrimination as adolescents get older.

### Reaction times

There was a significant main effect of Block (b_Block2 =−18.98, b_Block3 =−22.02, *χ*^2^(2) = 11.52, *p* = .003), such that RTs tended to get faster across task blocks. There was a significant main effect of Stimulus Type (b_CS+_nr_ = 19.87, b_CS−= 4.26, *χ*^2^(2) = 9.77, *p* = .008), such that RTs for the CS+_r_ tended to be faster than RTs for the CS+_nr_ (*Z* = 3.02, *p* = .003) but not the CS− (*Z* = .55, *p* = .581). There was a significant Stimulus Type × Block interaction (b_CS+_nr__Block2 = 21.29, b_CS−_Block2 = 30.66, b_CS+_nr__Block3 = 57.72, *χ*^2^(3) = 15.51, *p* = .001), such that RTs were faster for the CS+_r_ Block 3 compared with the CS+_nr_ Block 3 (*Z* = 4.60, *p* < .001) and CS− Block 2 (*Z* = 2.67, *p* = .007). Broadly, these results provide behavioral evidence that individuals discriminated between the stimuli.

### Functional connectivity (gPPI) analyses

#### Omnibus test of task effects

There was a significant effect for the omnibus contrast (F(8,32) = 2.32, *p* = .044), indicating that connectivity between the NAcc and Threat Network differed significantly as a function of condition (i.e., stimulus/block combinations) during the task. Follow-up ROI-specific analyses found a significant association between the NAcc and bilateral amygdala (F(8,32) = 2.28, *p* = .046) and the dACC (F(8,32) = 2.51, *p* = .031), but not the NAcc and the bilateral hippocampus (F(8,32) = 1.45, *p* = .214), bilateral insula (F(8,32) = 1.09, *p* = .393), or vmPFC (F(8,32) = 0.74, *p* = .653). To better interpret these findings, multilevel model results are reported below.

#### Multilevel modeling

##### Bilateral amygdala connectivity

**Figure 2.**
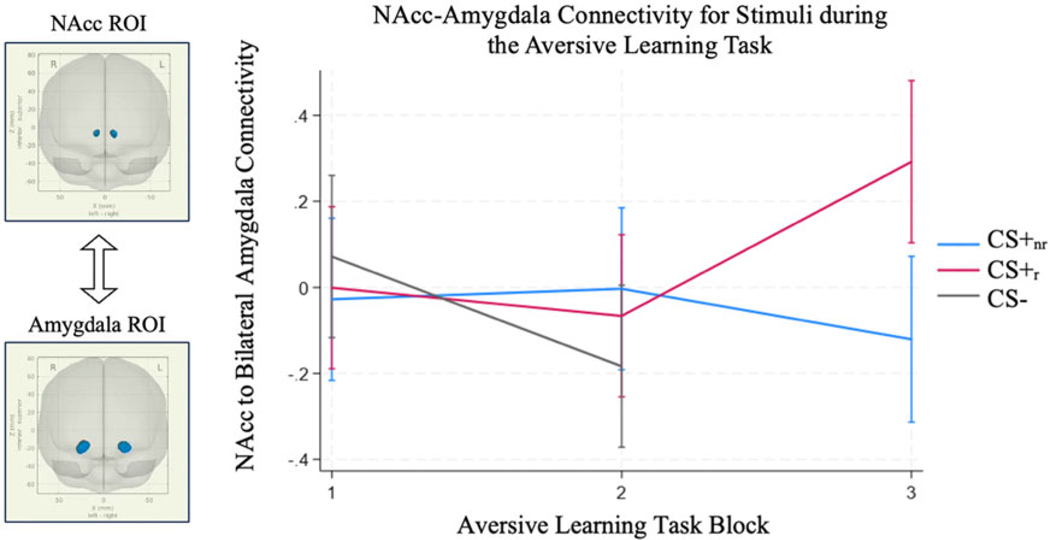
NAcc-amygdala functional connectivity differentiated stimuli by the end of the task, such that connectivity tended to increase for the CS+_r_ but decrease for the CS+_nr_ and the CS−.

The full model significantly explained variance in connectivity between the NAcc and bilateral amygdala (*χ*^2^(10) = 18.49, *p* = .047). There was an effect nearing significance for a Stimulus Type × Block interaction(b_CS+_nr__Block2 = .09,b_CS−_Block2 =−.19,b_CS+_nr__ Block3 =−.39, *χ*^2^(4) = 8.69, *p* = .069). Follow-up analyses found a significant simple main effect of Block for the CS+_r_ (b_Block2 =−.07, b_Block3 = .29, *χ*^2^(2) = 7.59, *p* = .023; Figure 2), a marginal effect for the CS− (b_Block2 =−.25, *χ*^2^(1) = 3.41, *p* = .065), and no effect for the CS+_nr_ (b_Block2 = .02, b_Block3 =−.09, *χ*^2^(2) = 1.07, *p* = .586). Pairwise comparisons similarly showed significantly greater connectivity for the CS+_r_ by the end of the task compared with earlier CS+_r_ blocks and compared with CS+_nr_ or CS− blocks (Table 2).

##### dACC connectivity.

The full model did not significantly explain variance in NAcc-dACC connectivity (*χ*^2^(10) = 11.37, *p* = .330). There was not a significant Stimulus Type × Block interaction (b_CS+_nr__Block2 = .04, b_CS−_Block2 =−.13, b_CS+_nr__Block3 = .36, *χ*^2^(4) = 3.89, *p* = .421).

#### Association with trait anxiety and depression symptoms

##### NAcc-amygdala connectivity

**Figure 3.**
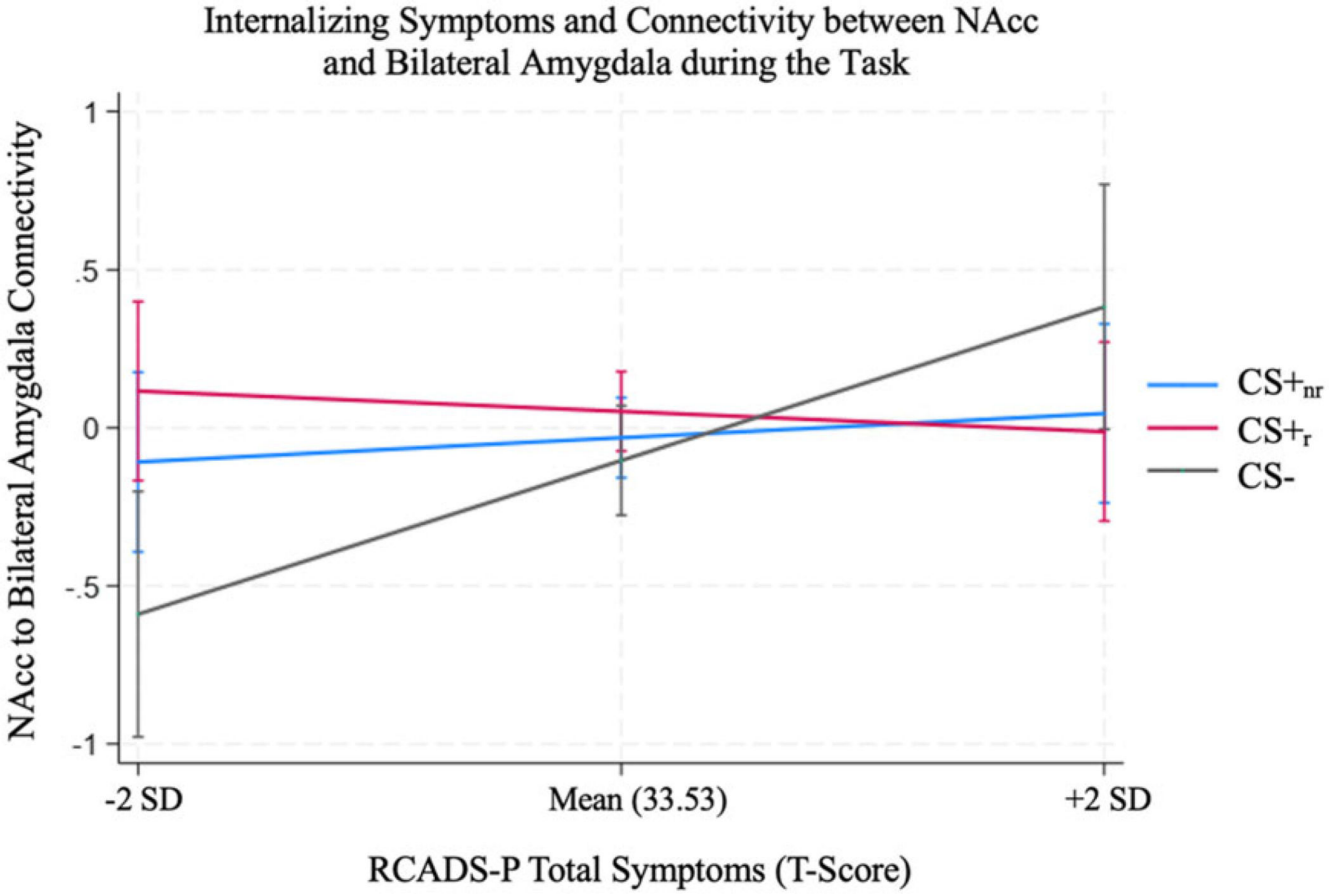
Greater internalizing symptoms were associated with greater NAcc-amygdala functional connectivity for the CS−, but not CS+_r_ or CS+_nr_, during the task.

There was no significant main effect of RCADS-P in predicting connectivity between the NAcc and bilateral amygdala (*b* = .01, *Z* = 1.43, *p* = .154). There was a significant RCADS-P × Stimulus Type interaction in predicting connectivity between the NAcc and bilateral amygdala (b_CS+_nr__RCADS = .01, b_CS −_RCADS = .04, *χ*^2^(2) = 6.37, *p* = .041), such that participants with greater trait anxiety and depression symptoms showed stronger positive connectivity for the CS− but not the CS+_r_ or CS+_nr_ (Figure 3).

##### Reaction time

There was a significant effect of RCADS-P over and above Age, Stimulus Type, Block, and Sex (*b* =−2.58, *Z* =−2.33, *p* = .020), such that participants with greater symptoms exhibited faster RTs. There was also a significant RCADS-P × Stimulus Type interaction (b_CS+_nr__RCADS =−2.66, b_CS−_RCADS =−.58, *χ*^2^(2) = 7.50, *p* = .024), such that participants with greater symptoms showed faster RTs specifically for the CS+_nr_, but not the CS+_r_ or CS−.

#### Exploratory analyses of age effects

##### Bilateral amygdala connectivity

**Figure 4.**
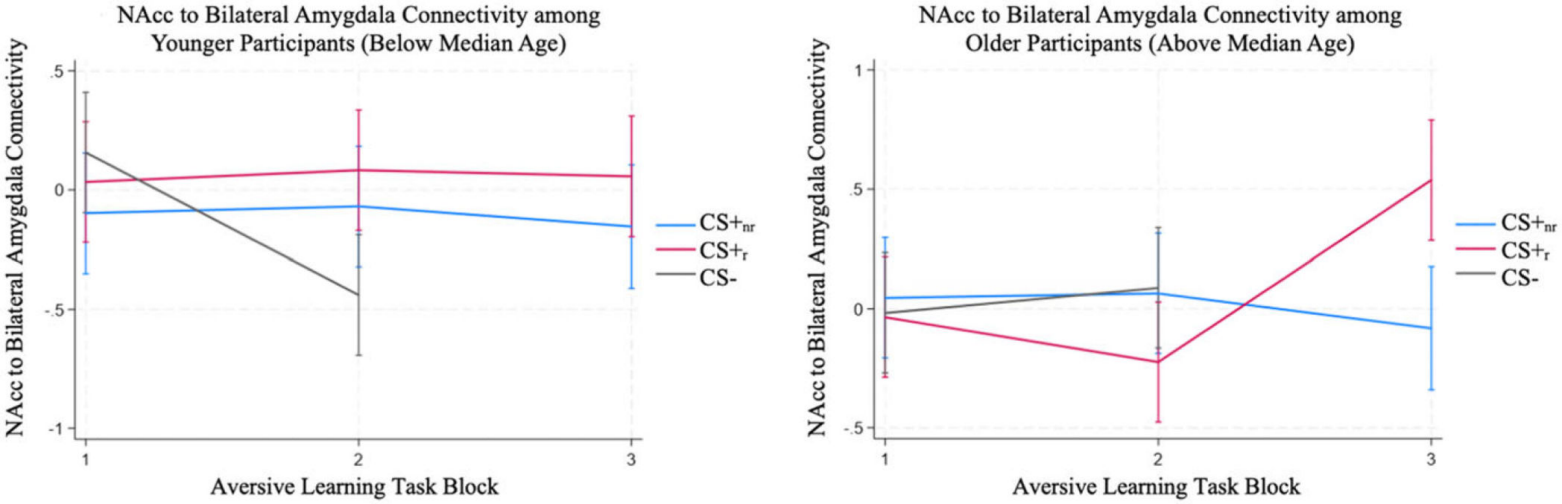
NAcc-amygdala functional connectivity during the task among participants younger (left) or older (right) than the median age in the sample (14.62 years). Age is shown categorically for interpretability. All analyses were conducted on continuous measures of age.

There was no significant main effect of Age (*b* = .02, *Z* = 1.44, *p* = .150) or Age × Stimulus Type interaction (b_CS+_nr_ _Age =−.01, b_CS−_Age = .02, *χ*^2^(2) = 1.87, *p* = .392) in predicting NAcc-amygdala connectivity during the task. There was a significant Age × Stimulus Type × Block interaction in predicting NAcc-amygdala connectivity during the task (b_CS+_nr__Block2_Age = .04, b_CS−_Block2_Age = .17, b_CS+_nr__Block3_Age = −.07, *χ*^2^(3) = 16.87, *p* = .001), such that connectivity for the CS+_r_ increased for older but not younger participants, and connectivity for the CS− decreased for younger but not older participants (Figure 4).

##### dACC connectivity

There was no significant main effect of Age (*b* = .00, *Z* = 0.30, *p* = .734), Age × Stimulus Type interaction (b_CS+_nr_ _Age =−.01, b_CS−_Age = .00, *χ*^2^(2) = .17, *p* = .917), or Age × Stimulus Type × Block (b_CS+nr_Block2_Age = .04, b_CS−_Block2_Age = .09, b_CS+nr_Block3_Age =−.01, *χ*^2^(3) = 3.33, *p* = .344) interaction in predicting NAcc-dACC connectivity during the task.

##### Reaction time

There was a significant main effect for Age in predicting RT (*b* =−6.81, *Z* =−3.95, *p* < .001), such that older participants had faster RTs than younger participants throughout the task. There was no significant Age × Stimulus Type interaction in predicting RTs (b_CS+_nr_ _Age =−.47, b_CS−_Age = 1.15, *χ*^2^(2) = .66, *p* = .719), suggesting that older participants had faster RTs regardless of stimulus type.

## Discussion

This study evaluated functional connectivity between reward and threat neurocircuitry among adolescents during an aversive learning task that included a condition with an opportunity to escape threat. Over the course of the task, we found that functional connectivity between the NAcc and bilateral amygdala became increasingly positive for an escapable aversively reinforced stimulus (CS+_r_), stable and nonsignificant (i.e., noncorrelated activity) for the same stimulus when it was not reinforced (CS+_nr_), and marginally negative for a stimulus that was never reinforced (CS−). These results suggest that, among adolescents, NAcc-amygdala connectivity may play an important role during escape from threats but not during safety. To our knowledge, the present study is the first to highlight a NAcc-amygdala circuit during adaptive threat responding in adolescent humans. Furthermore, individual differences in trait anxiety and depression symptoms were associated with more positive NAcc-amygdala connectivity for the CS−, which may reflect a link between adolescent psychopathology and aberrant safety learning. Collectively, these findings provide a novel perspective on how threat and reward neurocircuitry support adaptive behavior during adolescent development.

### Reward processes in threat learning

Reward processes are considered central to survival behaviors, such as escape and avoidance of danger, and may work in part by activating reward-related neurocircuitry that supports positive emotional experiences (e.g., relief) to reinforce escape or avoidance (Rosenberg, Barnes-Horowitz, et al., 2024). Results from the present study support this notion, as the NAcc (frequently considered in the context of reward paradigms) and amygdala (frequently considered in the context of threat paradigms) showed greater coordination during escape from threat versus during safety.

These findings add to a growing literature showing recruitment of canonical reward neurocircuitry in the context of threat learning paradigms. For example, rodent studies have implicated the NAcc in (1) discrimination of threat and safety (Ray et al., 2020, 2022; Stelly et al., 2019), (2) prediction error learning during threat learning (Delgado et al., 2008; Oleson et al., 2012), and (3) responding to the termination of pain (i.e., negative reinforcement) (Navratilova et al., 2012). Human studies have similarly shown that the NAcc signals more strongly during instrumental avoidance learning than fear extinction (Boeke et al., 2017; Garrison et al., 2013), suggesting that the NAcc may play a role during the execution of motivated behaviors (e.g., escape or avoidance) (Delgado et al., 2009) but not during more passive learning of threat or safety associations (e.g., acquisition or extinction). Indeed, rodent and human studies have highlighted projections from the basolateral amygdala to the NAcc that support goal-directed actions such as instrumental avoidance and escape behavior (e.g., Ramirez et al., 2015; for a review, see LeDoux & Daw, 2018). The present study demonstrates a similar NAcc-amygdala circuit is evident during escape from threat among a sample of typically developing adolescent humans.

### Developmental significance of reward-threat interactions

While the NAcc and associated risk-taking behaviors are central to many neuroscientific accounts of adolescence (Ernst et al., 2006; Ernst, 2014; Richards et al., 2012), relatively little work has considered the role that the NAcc may play in threat processes during adolescence. However, as in reward paradigms, threat-related activation of the NAcc also increases during pubertal maturation and is theorized to relate to positive emotions that are triggered by successfully escaping danger (Spielberg et al., 2014). The exploratory analyses of age effects complement and extend this prior work, as older versus younger adolescents exhibited greater threat-related engagement of a NAcc-amygdala circuit for the CS+_r_. Older adolescents also exhibited faster RTs during the aversive learning task, although this effect was not specific to the CS+_r_. Together, these results suggest that threat-related NAcc-amygdala connectivity strengthens during adolescence in support of adaptive threat learning.

These findings may be integrated within the broader literature on NAcc engagement during adolescent development. For example, it is possible that increasing NAcc engagement during adolescence not only promotes approach behavior in potentially threatening situations but also serves a protective role by enabling the successful deployment of amygdala-mediated avoidance or escape behaviors in threatening situations. If so, this may have implications for existing conceptualizations of “positive risk-taking” in adolescence, which have traditionally considered the relative benefits and costs associated with the risky behavior (Duell & Steinberg, 2019, 2021). For example, positive risk-taking behaviors (e.g., initiating a friendship with a new classmate or trying out for a sport) during adolescence may additionally open up new opportunities for adolescents to learn that (1) they are capable of successfully escaping danger, or (2) a given situation is not as dangerous as was originally predicted (i.e., fear extinction), processes which are considered anxiolytic (Craske et al., 2022). Rewarding positive emotions, such as relief or thrill, may draw upon similar neurocircuitry and reinforce positive risk-taking behaviors, ultimately promoting adaptive threat learning during adolescence.

### Implications for developmental psychopathology

The present study highlighted associations between adolescent internalizing symptoms and elevated NAcc-amygdala connectivity during safety learning. These results are consistent with existing models of developmental psychopathology, wherein inaccurate discrimination of threat and safety cues is considered a hallmark of emerging anxiety symptoms (Britton et al., 2011) and further relates to transdiagnostic symptom dimensions, such as anhedonia, from adolescence into young adulthood (Rosenberg, Young, et al., 2024). If elevated NAcc-amygdala coordination occurs in safe contexts for some adolescents, those adolescents may be more likely to activate motivated behaviors designed to circumvent threats (e.g., avoidance or escape). For example, we found that greater internalizing symptoms were also associated with faster RTs for the safe CS+_nr_, which may reflect similar inaccuracies in threat and safety discrimination. Such a possibility deserves careful consideration given that contemporary models of clinical anxiety emphasize over-reliance on behavioral avoidance (e.g., in safe contexts), as this can limit opportunities for fear extinction (Beckers et al., 2023; Graham & Milad, 2011; Pittig et al., 2018), increase fear renewal following extinction (Arnaudova et al., 2017), and preserve or even increase conditional fear responses that further reinforce instrumental avoidance behaviors (Lovibond et al., 2009; Pittig et al., 2020). Additional research is needed to test these possibilities.

Importantly, as the peak onset for anxiety and depressive disorders occurs at the same time that NAcc responsivity tends to peak during adolescence (Kessler et al., 2007, 2012; Merikangas et al., 2010; Paus et al., 2008; Powers & Casey, 2015; Rapee et al., 2019), it is possible that individual differences in safety-related NAcc expression may inform which individuals are most likely to develop symptoms. Indeed, greater NAcc activation during active avoidance has been shown to correlate with anxiety symptoms among adults (Levita et al., 2012), suggesting that emerging aberrances in threat-related NAcc activity may precede anxiety symptoms long-term. Healthy brain and behavioral development during adolescence may therefore involve a tenuous balance across systems responsible for processing rewards and threats to promote adaptive responding in the face of true danger without promoting overreliance on avoidance strategies for managing fear. Future research should evaluate (1) how the NAcc-amygdala circuit relates to safety learning among clinically anxious adolescents, (2) if NAcc-amygdala connectivity during adolescence predicts anxiety symptoms into adulthood, and (3) if emerging symptoms of anxiety become increasingly associated with aberrant NAcc-amygdala connectivity as adolescents get older.

While the present study did not consider what factors drive individual differences in NAcc-amygdala threat processes, it stands to reason that environmental exposures may strongly influence how reward and threat neurocircuitry interact during adolescent development. For example, experiences of early life adversity have been shown to alter both threat and reward circuitry (McLaughlin et al., 2019), particularly recruitment of the amygdala and ventral striatum (Fareri & Tottenham, 2016). It is thought that these alterations confer greater risk for psychopathology, in part, due to their association with aberrances in threat and reward learning (McLaughlin et al., 2019). For example, escapable threats extinguish more readily than inescapable threats, suggesting that uncontrollable environmental factors during development may be especially likely to confer increased risk for psychopathology (Cohodes et al., 2023; Hartley et al., 2014). Future research is needed to examine if early life adversity alters recruitment of the NAcc-amygdala circuit during aversive learning, thereby biasing behavioral responses toward maladaptive responses (e.g., escape or avoidance in safe contexts).

### Limitations and future directions

The present study should be interpreted in light of several limitations. First, results from the present study should be replicated in a larger sample of adolescents across a wider variety of aversive learning paradigms – for example, given the “social reorientation” that occurs during adolescence, it would be especially informative to examine approach and avoidance behavior around valenced social stimuli. Second, while the majority of participants were within an age range typically overlapping with conventional definitions of adolescence, the age range of our sample spans early adolescence to early adulthood. Longitudinal research with an explicit focus on age (rather than exploratory cross-sectional analysis of age as a moderator) is needed to evaluate developing threat and reward neurocircuitry, as this could more definitively test if the NAcc-amygdala aversive learning circuit emerges continuously throughout the adolescent window. Third, although prior studies suggest activation of the NAcc and amygdala both increase during instrumental avoidance (LeDoux & Daw, 2018), the present study was not optimized to evaluate directionality of brain connectivity. Fourth, as individual differences in fear and threat processes are most detectable in studies with ambiguous stimuli (i.e., “strong situation effect,” see Lissek et al., 2006), it is possible that symptom associations would not be observed in other unambiguous task designs (e.g., inclusion of CS+_r_ blocks without CS+_nr_ blocks). Finally, as anxiety and depression symptoms were assessed at trait levels, the clinical applicability of these results should be interpreted with caution. Additional research is needed to replicate and extend these findings among clinically anxious or depressed youth and among individuals at risk for psychopathology (e.g., those exposed to early life adversity).

## Conclusion

In conclusion, the present study evaluated functional connectivity between the NAcc and canonical threat neurocircuitry among adolescents during an aversive learning task. Results indicated that greater connectivity between the NAcc and bilateral amygdala was associated with escapable threat versus safety. Trait-level anxiety and depression symptoms were associated with greater connectivity between the NAcc and bilateral amygdala during safe blocks, suggesting persistent engagement of defensive neurocircuitry as a mechanism of emerging psychopathology among adolescents. Additional research is needed to replicate and extend these findings in other samples and with other paradigms.

## Supplementary Material

1

## Figures and Tables

**Table 1.** Participant demographics in the study

Parent-Reported Participant Race	Total Number	Percentage of Sample
African American or Black	7	14.89%
American Indian or Alaska Native	0	0%
Asian American	6	12.77%
Caucasian	21	44.68%
Mixed	5	10.64%
Native Hawaiian or Other Pacific Islander	1	2.13%
No Response Given	3	6.38%
Other	4	8.51%
Parent-Reported Participant Ethnicity	Total Number	Percentage of Sample
Hispanic or Latino	5	10.64%
Not Hispanic or Latino	42	89.36%
Parent Reported Household Income	Total Number	Percentage of Sample
Less than $10,000 per year	4	8.51%
$10,0001–$25,000 per year	1	2.13%
$25,001–$40,000 per year	0	0.00%
$40,001–$55,000 per year	2	4.26%
$55,001–$70,000 per year	6	12.77%
$70,001–$85,000 per year	2	4.26%
$85,001–$100,000 per year	6	12.77%
$100,001–$150,000 per year	4	8.51%
$150,001–$200,000 per year	11	23.40%
Greater than $200,000 per year	9	19.15%
Not Reported	2	4.26%
Parent Reported Education (Self)	Total Number	Percentage of Sample
Some high school	0	0.00%
High school degree	0	0.00%
Some college	6	12.77%
Community college / two-year degree	1	2.13%
Four-year college degree	7	14.89%
Some graduate school	4	8.51%
Master’s degree	16	34.04%
Doctoral degree	9	19.15%
Professional degree	0	0.00%
Other	2	4.26%
Not Reported	2	4.26%
RCADS-P Symptom T-Score (Mean = 33.53, SD = 5.96)	Total Number	Percentage of Sample
Less than 20	0	0.00%
21–30	12	25.53%
31–40	18	38.30%
41–50	3	6.38%
Greater than 50	1	2.13%

**Table 2.** Covariate results and significant pairwise contrasts among stimulus/block pairs in the NAcc-amygdala connectivity analyses

Covariates	b and 95% CI	Z-Statistic	*p*
Age	0.02 [ − .01, .04]	1.46	0.145
Sex	0.00 [ − .14, .14]	0.04	0.967
Mean Framewise Displacement	0.53 [ − .23, 1.29]	1.36	0.175
Stimuli	Contrast	Z-Statistic	*p*
CS+_r_ Blocks	CS+_r3_ > CS+_r1_	2.15	0.013
	CS+_r3_ > CS+_r2_	2.64	0.008
CS+_r3_ vs CS+_nr_	CS+_r3_ > CS+_nr1_	2.35	0.019
	CS+_r3_ > CS+_nr2_	2.17	0.03
	CS+_r3_ > CS+_nr3_	3.00	0.003
CS+_r_ vs CS−	CS+_r3_ > CS−_2_	3.50	< .001

## Footnotes

1
https://web.conn-toolbox.org/fmri-methods/preprocessing-pipeline
