# Supplementary material for: Functional connectivity between the nucleus accumbens and amygdala underlies avoidance learning during adolescence: Implications for developmental psychopathology
Source: Dev Psychopathol. Author manuscript; Available in PMC 2025 Sep 5. (PMC11936845; doi:10.1017/S095457942400141X)
Supplement: 1 [file NIHMS2018456-supplement-1.docx]

**Supplement**

***Seed-to-Voxel Analyses***

Supplementary analyses applied a seed-to-voxel approach to evaluate NAcc connectivity across the whole brain during the different task blocks. We implemented gPPI in the CONN Toolbox applying a voxel threshold *p*-unc < .001 and cluster threshold *p*-FWE < .05. An analysis of CS+_r_ > CS- identified no significant clusters. An analysis of CS+_nr_ > CS- identified one significant cluster centered on MNI coordinates [-6 12 -16] overlapping with voxels in the subcallosal cortex (*p*-FWE = .005).


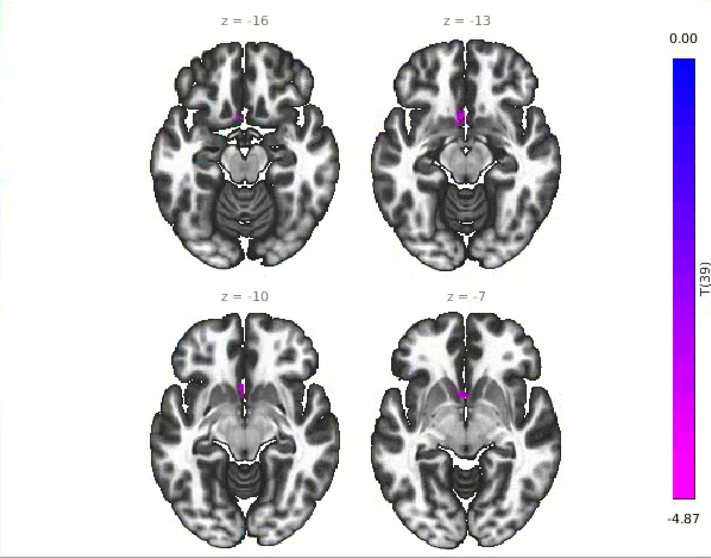


Fig. S1: Visual representation of significant seed-to-voxel results for the CS+_nr_ > CS- contrast.

***Activation Analyses***

Supplementary analyses evaluated activation for each ROI implicated in the main analyses. Using the same multilevel modeling approach detailed in the main paper, we analyzed a three-way Stimulus Type x Block x ROI interaction in predicting brain activation during the task (covarying for Age, Sex, and Mean Framewise Displacement). There was not a significant Stimulus Type x Block x ROI interaction (b=-.205, ꭓ^2^(3)=1.52, *p*=.678), such that there were no significant pairwise differences in activation between the NAcc and amygdala for any of the stimulus/block combinations. Plots of average activation across task blocks are depicted below for each ROI, separated by stimulus type:


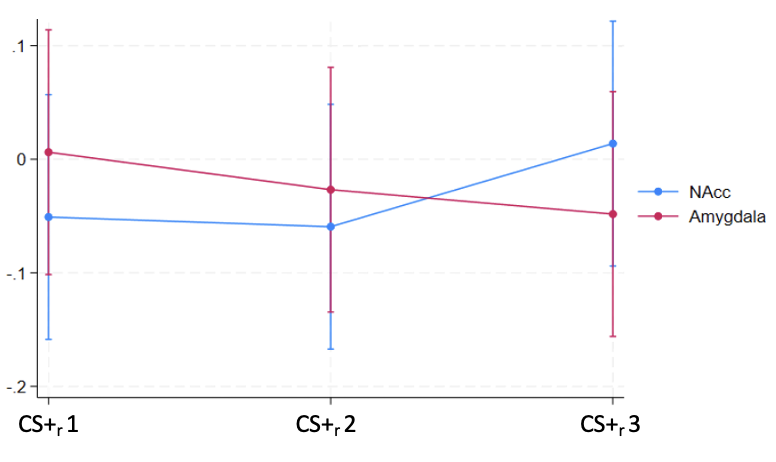


Fig. S2: Average activation of NAcc and amygdala for CS+_nr_ blocks.


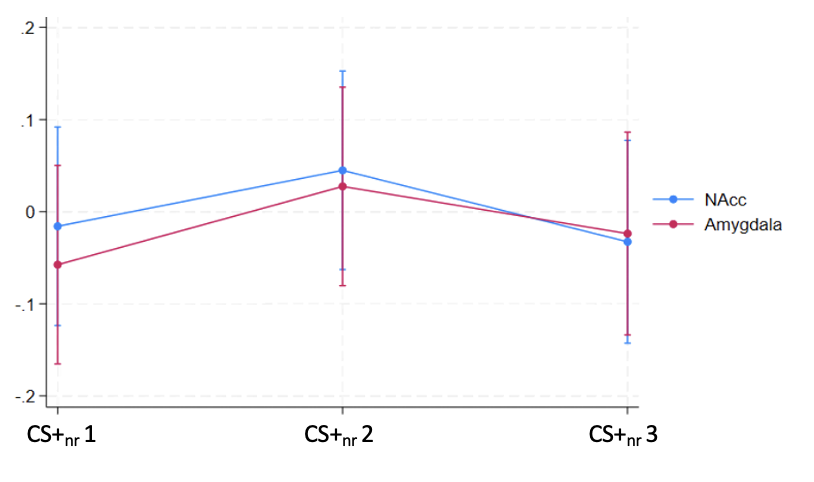


Fig. S3: Average activation of NAcc and amygdala for CS+_r_ blocks.


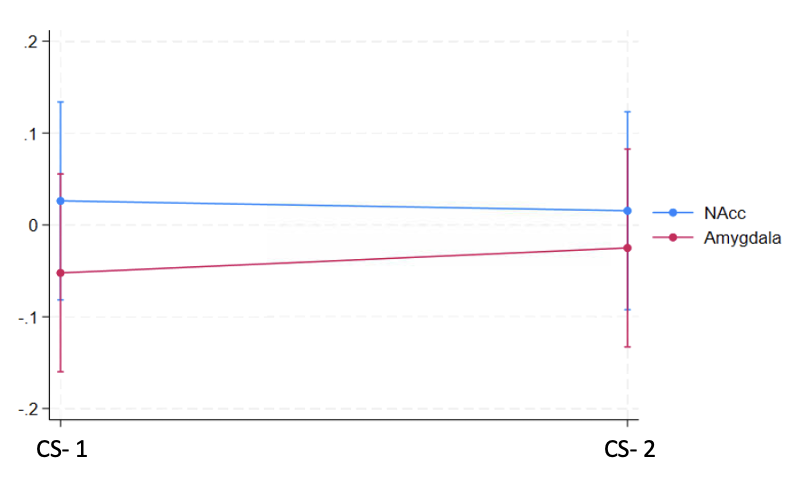


Fig. S4: Average activation of NAcc and amygdala for CS- blocks.
